# Optimization and validation of a reversed-phase high performance liquid chromatography method for the measurement of bovine liver methylmalonyl-coenzyme a mutase activity

**DOI:** 10.1186/1471-2091-14-25

**Published:** 2013-10-16

**Authors:** Bazoumana Ouattara, Mélissa Duplessis, Christiane L Girard

**Affiliations:** 1 Dairy and Swine Research and Development Centre, Agriculture and Agri-Food Canada, 2000 College Street, Sherbrooke, Quebec J1M 0C8, Canada

**Keywords:** Methylmalonyl-CoA mutase, Liver, Cattle, Dairy cow, Succinyl-CoA, RP-HPLC

## Abstract

**Background:**

Methylmalonyl-CoA mutase (MCM) is an adenosylcobalamin-dependent enzyme that catalyses the interconversion of (2R)-methylmalonyl-CoA to succinyl-CoA. In humans, a deficit in activity of MCM, due to an impairment of intracellular formation of adenosylcobalamin and methylcobalamin results in a wide spectrum of clinical manifestations ranging from moderate to fatal. Consequently, MCM is the subject of abundant literature. However, there is a lack of consensus on the reliable method to monitor its activity. This metabolic pathway is highly solicited in ruminants because it is essential for the utilization of propionate formed during ruminal fermentation. In lactating dairy cows, propionate is the major substrate for glucose formation. In present study, a reversed-phase high performance liquid chromatography (RP-HPLC) was optimized and validated to evaluate MCM activity in bovine liver. The major aim of the study was to describe the conditions to optimize reproducibility of the method and to determine stability of the enzyme and its product during storage and processing of samples.

**Results:**

Specificity of the method was good, as there was no interfering peak from liver extract at the retention times corresponding to methylmalonyl-CoA or succinyl-CoA. Repeatability of the method was improved as compared to previous RP-HPLC published data. Using 66 μg of protein, intra-assay coefficient of variation (CV) of specific activities, ranged from 0.90 to 8.05% and the CV inter-day was 7.40%. Storage and processing conditions (frozen homogenate of fresh tissue vs. fresh homogenate of tissue snapped in liquid nitrogen) did not alter the enzyme activity. The analyte was also stable in liver crude extract for three frozen/thawed cycles when stored at -20°C and thawed to room temperature.

**Conclusions:**

The improved method provides a way for studying the effects of stages of lactation, diet composition, and physiology in cattle on MCM activity over long periods of time, such as a complete lactation period. Interestingly, this sensitive and accurate method could benefit the study of the cobalamin status in experimental studies and clinical cases.

## Background

Methylmalonyl-CoA mutase (MCM) is a bacterial and vertebrate adenosylcobalamin-dependent enzyme that catalyses the interconversion of (2R)-methylmalonyl-CoA to succinyl-CoA [1]. In some bacteria, such as *Propionibacterium shermanii*, MCM is important for the fermentation of pyruvate to propionate [2]; linking the production of propionate from succinate in a reverse metabolic pathway [3]. In higher animals, including human, MCM is required for the metabolism of methylmalonyl-CoA [4]. In humans, a deficit in activity of MCM, due to an impairment of intracellular formation of adenosylcobalamin and methylcobalamin, respective cofactors for the MCM and methionine synthase, results in a wide spectrum of clinical manifestations (reviewed by Carrillo-Carrasco [5]). Methylmalonyl-CoA is produced by catabolism of odd-chain fatty acids and some amino acids such as valine, isoleucine and methionine. This metabolic pathway has a high flux level in ruminants for gluconeogenesis from large amounts of propionate formed during ruminal fermentation [6]. Therefore, in ruminants, MCM plays a major role in energy production via succinyl-CoA, an essential step for its entry in Krebs cycle [7]. In dairy cows, increasing affinity of MCM for its cofactor increases gluconeogenesis [8]. Owing to its importance, determining the activity of MCM is the subject of abundant literature [8-28]. Several methods based on the quantification of succinyl-CoA produced from methylmalonyl-CoA, have been used to study MCM activity. The radioassay based on the permanganate oxidation of DL[CH3-^14^C]methylmalonyl-coenzyme A is the most commonly used. Unfortunately, sensitivity and reproducibility of the radiometric method are low [7,29]. To overcome these problems, a spectrophotometric assay has been developed to allow a more accurate estimation of MCM kinetic parameters [7,8] however, spectrophotometric methods are more appropriate for studying the activity of purified enzyme. An electrophoretic separation method for MCM activity using surfactant for separation was also proposed [30]. However, the injection of high-concentration surfactant into the electrophoretic capillary may decrease both the separation efficiency and the reproducibility [31]. Other chromatographic techniques were also used to measure MCM activity [19,29]. However the existing liquid chromatographic methods suffer from a lack of validation data to support their routine use. In the present work, a RP-HPLC method was optimized and validated to measure MCM activity in bovine liver. The analyte stability in regard to tissue storage conditions was assessed. The performance of the method was evaluated by measuring holo and total MCM activity in the liver of six cows. Coefficients of variation (CV) and standard deviation of repeated analyses, as well as system suitability parameters were determined. The major aim of the study was to describe the conditions to optimize reproducibility of the method.

## Methods

### Reagents and chemicals

Methylmalonyl-coenzyme A (lithium salt), succinyl-coenzyme A (sodium salt), 5′-deoxyadenosylcobalamin (AdoCbl), phosphoric acid, trichloroacetic acid (TCA), tris-base, sodium phosphate monobasic and sodium phosphate dibasic were purchased from Sigma-Aldrich (Canada Ltd, Oakville, ON, CANADA). HPLC-gradient grade methanol was obtained from Fisher (Fair Lawn, NJ, USA). One mL-TB (Tuberculin) syringes were purchased from BD (Franklin Lakes, NJ, USA), PVDF (polyvinylidene difluoride) syringe filters (units) 0.45 μm were manufactured by National Scientific Company (Rockwood, TN, USA) and 1.5 mL boil-proof microtubes were distributed by Ultident Scientific (Montréal, QC, Canada). Filter sheets 0.2 μm were manufactured products of Pall Corporation (Ann Arbor, MI, USA).

### Sample preparation

Bovine liver was purchased from a slaughterhouse (Viande Giroux, East Angus, QC, Canada), collected immediately after slaughter and transported on ice to the laboratory within 50 minutes. Upon arrival at the laboratory, a portion of the liver was homogenized on ice in 4.5 volumes (w/v) of cold distilled water. The homogenates were centrifuged at 750 *g* for 10 minutes at 4°C. The supernatants were then aliquoted (1 mL) and stored in microcentrifuge tubes at –80°C. The remaining hepatic tissue was cut in pieces, immediately frozen into liquid nitrogen and stored at -80°C until use. On the day of analysis, a small volume (~30 μL) was used to measure protein concentration by the method of Bradford using bovine serum albumin as standard (Bio-Rad protein assay kit, Bio-Rad Laboratories (Canada) Ltd, Mississauga ON, Canada). Then, the remainder of the homogenate was diluted with distilled water to obtain the chosen protein concentration for the assay. Hepatic tissue was also obtained by liver biopsy performed on 6 lactating dairy cows, 64 ± 3 days after calving, under local anesthesia and ultrasound guidance to minimize the hemorrhagic risks as described by Graulet et al. [8]. The procedure was approved by the Institutional Committee on Animal Care of the Dairy and Swine Research and Development Centre, Agriculture and Agri-Food Canada, Sherbrooke, QC, Canada. Tissues were immediately frozen into liquid nitrogen and stored at -80°C until use. Homogenates from frozen tissues (biopsies from dairy cows and tissue from the slaughterhouse) were prepared as described above.

### Preparation of standards

A stock solution of methylmalonyl-CoA (1 mM) was prepared in 300 mM of tris-phosphate buffer, pH 7.5; this solution was aliquoted and kept at –30°C. Thus, calibration solutions of methylmalonyl-CoA could be prepared by diluting the stock solution in 100 mmol/L phosphate buffer, pH 4.0. The succinyl-CoA calibration solutions (stock, 500 μM) were prepared in 200 mmol/L phosphoric acid, pH 1.8, aliquoted and stored at –30°C. A solution of AdoCbl (1 mM) for the determination of total enzyme activity was also prepared in distilled water, protected against light inactivation and stored at –30°C.

### Liquid chromatography

The HPLC analyses were carried out on an Agilent technology system consisting of two proStar 210 pumps, 320 UV detector and 420 AutoSampler. The Agilent Poroshell 120 EC-C18, 2.7 μm, 3.0 mm × 100 mm threaded column was heated at 40°C with a HPLC column heater Croco-cil^®^. The assay reaction mixture for total MCM activity contained, in a total volume of 150 μL, 16 to 333 μg of liver homogenate protein (60 μL), 30 μL of AdoCbl (1 mM), and 60 μL of methylmalonyl-CoA (1 mM). The liver homogenate was first incubated with AdoCbl for 5 minutes at 37°C before initiating the enzyme reaction by adding methylmalonyl-CoA. The tubes were again incubated at 37°C for 0 to 30 minutes and the reaction was stopped by adding 50 mL of trichloroacetic acid (TCA; 100 g/L). The tubes were centrifuged at 13 000 *g* for 1 min. The supernatant was collected and filtered in amber glass vials through a 0.45 μm syringe-operated filter units. A volume of 20 μL of sample was injected in the HPLC system. Blanks were included in every run.

The reverse phase column was equilibrated with 56% solvent A (100 mM acetic acid in 100 mM of sodium phosphate buffer, pH 7.0) and 44% solvent B (18% v/v methanol in solvent A). The method consisted in a linear methanol gradient: 0 – 3 min (44% solvent B); 3 – 9 min (44 – 75%); 9 – 12 min (75 – 100%); 12 – 17 min (100 – 44%); and 17 – 35 min (44%) with a flow rate of 0.2 mL/min. The solvent A was filtered through 0.2 μm membrane. The compounds were detected at 254 nm.

### Validation of the method

Method validation was performed according to guidelines set by the United States Food and Drug Administration (FDA) for bioanalytical method validation [32]. Hence, linearity, specificity, low limit of quantitation (LLOQ), recovery, intra- and inter-day accuracy and precision, and stability of the analyte during sample storage and processing procedures were determined.

#### ***Linearity and sensitivity***

The lowest standard on the calibration curve has been determined by testing a range from 0.48 to 500 μM. Each concentration was prepared in duplicate and the test was repeated once. Calibration curves were built by plotting peak areas versus concentrations of methylmalonyl-CoA or succinyl-CoA, and the regression equations were calculated. Low limits of detection (LOD) were determined when electropherograms produced peaks with a signal-to-noise ratio (S/N) of 3 at 254 nm wavelength. The LLOQ were determined based on the S/N (> 5), CV (< 20%) and accuracy (> 80%). Linearity of the reaction according to enzyme concentrations obtained by different dilutions of the liver extract homogenates was also determined. The amounts of protein tested were: 16, 26, 33, 52, 66, 83, 104, 166, 208 and 333 μg. The linearity of the enzyme reaction versus incubation time (0, 5, 10, 15, 20, 25, 30 and 40 min) was also measured.

#### ***Specificity***

The specificity of the method for dilutions of methylmalonyl-CoA and succinyl-CoA was tested on samples spiked and not spiked with known standards.

#### ***Method precision and recovery***

The precision of the instruments was checked using samples injected repeatedly (n = 45). Forty-five replicates of homogenates providing 66 μg of protein were assayed over nine days. The intra- and inter-assay precisions were determined using the CV. Separate runs of three levels of standards (Low = 7.8 μM; Middle = 62.5 μM and High = 500 μM) were made. The accuracy (%) of the method was expressed by [(Measured concentration)/(Nominal concentration)] × 100.

The recovery of succinyl-CoA was studied by addition of a known amount of standard (75 μM) to prequantified samples after stopping the reaction. Briefly, a volume of 50 μL of TCA was added to the reaction mixture to stop the reaction and after centrifugation, 170 μL of the supernatant was collected. This volume was completed to 200 μL by adding 30 μL of standard (stock, 500 μM). Recovery was performed by comparing the analytical results for extracted samples at the three concentrations tested (33, 66 and 166 μg of protein) with standards that represent 100% recovery. Recovery (%) = [(Measured concentration in spiked sample – Measured concentration in non-spiked sample)/Standard concentration] × 100.

#### ***Stability study***

The amounts of succinyl-CoA formed using frozen extracts prepared from fresh liver or extracts prepared from tissue snapped in liquid nitrogen were compared and the CV between the two modes of preparation was calculated. The percentages of difference between days of runs were also calculated as follow:% difference day_n_ vs day_n+1_ = [(Concentration on day_n_- Concentration on day_n+1_)/Concentration on day_n_)] × 100. These runs included two vials of a known concentration of the standard succinyl-CoA (75 μM) and two vials of standards (succinyl-CoA + methylmalonyl-CoA, 125 μM each) mixed to check the reliability of the method. The stability of standard and sample solutions when re-injected after being left at room temperature for more than 24 hours was evaluated to detect if the time spent in the autosampler affected the measurements.

### Pharmacokinetic study

As component of ongoing studies to validate the HPLC method for the evaluation of MCM activity in bovine liver extract, pharmacokinetics of MCM was studied. The kinetics of MCM was established by testing increasing concentrations of substrate, methylmalonyl-CoA: 6.25, 12.5, 25, 50, 100, 200 and 400 μM. The initial amount of protein (enzyme) and the time of incubation were 66 μg and 30 min respectively.

Holomutase and total specific activities were measured in liver tissue obtained by biopsy from six lactating dairy cows. Testing increasing concentrations of protein allowed us to find that using 30-40 μg of protein was sufficient to obtain reproducible results from these homogenates.

### Data analysis

Means, standard errors and CV were calculated using Excel software. In figures, values are means, with their standard errors (SE) represented by vertical bars.

## Results

### Method validation

#### ***Linearity and sensitivity***

**Figure 1:**
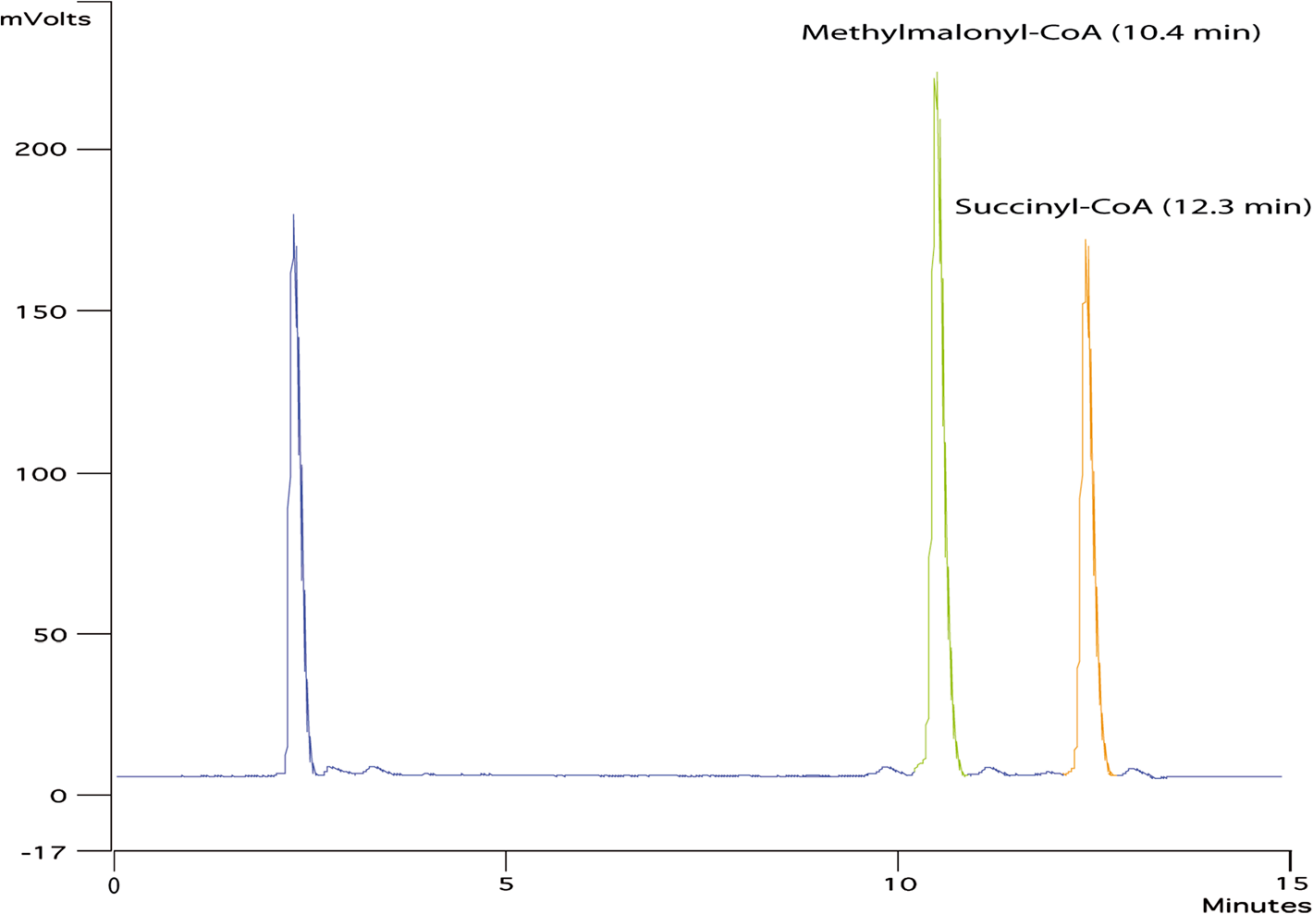
**Electropherograms of a solution of methylmalonyl-CoA and succinyl-CoA.** Chromatograms were obtained using 0.1 M sodium phosphate buffer (pH 7) containing 100 mM of acetic acid, as solvent A of the mobile phase, and 18% of methanol in solvent A being the solvent B. The column temperature was 40°C.

The lowest concentrations of succinyl-CoA and methylmalonyl-CoA measured with precision were respectively 6.74 μM and 15.33 μM (Table 1). However the LOD were about 1 μM for succinyl-CoA and 2 μM for methylmalonyl-CoA. Figure 1 shows the typical chromatogram of a mixture of methylmalonyl-CoA (RT = 10.4 min) and succinyl-CoA (RT = 12.3 min). Calibration curves were plotted from LLOQ of each standard to 500 μM. The CV for the seven calibration standard points of succinyl-CoA ranged from 0.15 to 10.38% with accuracy varying from 86 to 102.37% (Table 1). The curve of methylmalonyl-CoA (Additional file 1: Figure S1) had six points with CV of 0.10 to 1.34% and accuracy varying from 98.12 to 101.83% (Table 1).

**Table 1:** Limits of quantification of succinyl-CoA and methylmalonyl-CoA

**Succinyl-CoA**			
Nominal concentration (μM)	Mean calculated concentration (μM)	CV%n = 4	Accuracy%
7.81	6.74	10.38	86.33
15.62	15.35	1.22	98.28
31.25	30.72	1.20	98.32
62.5	63.98	1.65	102.37
125	125.71	0.40	100.57
250	249.47	0.15	99.79
500	497.58	0.34	99.52
**Methylmalonyl-CoA**			
Nominal concentration (μM)	Mean calculated concentration (μM)	CV%	Accuracy%
		n = 4	
15.62	15.33	1.34	98.12
31.25	31.20	0.10	99.85
62.5	63.65	1.28	101.83
125	125.55	0.31	100.44
250	249.47	0.15	99.79
500	495.82	0.59	99.16

The calculated concentrations are compared to nominal concentrations and the coefficients of variation (CV%) are presented. Solvent A of the mobile phase contained 0.1 M sodium phosphate buffer (pH 7) with 100 mM of acetic acid. Solvent B consisted of 18% methanol in solvent A. The column temperature was 40°C.

System suitability parameters such as theoretical plate number, relative retention time, tailing, selectivity and capacity factors between methylmalonyl-CoA and succinyl-CoA presented in Additional file 2: Table S1 were checked in order to verify the system, method and column performance. The resolution between methylmalonyl-CoA and succinyl-CoA is not less than 2 and tailing factor for each peak is not more than 2. The plate count is also greater than 2000.

**Figure 2:**
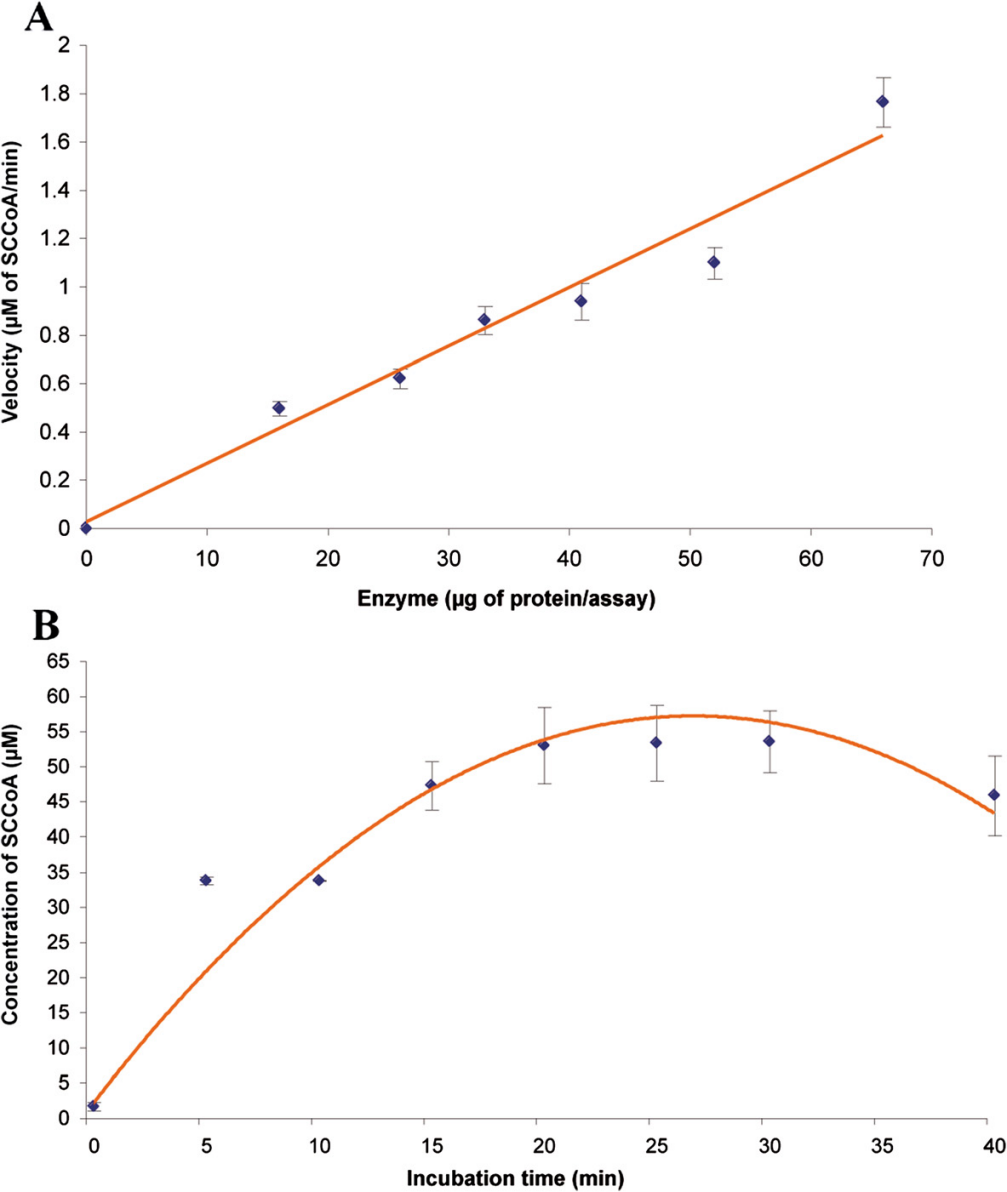
**Formation of succinyl-CoA according to protein amount or time of incubation. A)** Rate of formation of succinyl-CoA per min according to the amount of protein used in the assay after 30 min of incubation at 37°C. Each amount of protein was tested 4 times and the initial concentrations of AdoCbl and MMCoA were 200 μM and 400 μM respectively. **B)** Concentration of succinyl-CoA (μM) formed by 66 μg of protein at 37°C as a function of incubation time (expressed in minutes). The initial concentration of AdoCbl and MMCoA were 200 μM and 263 μM respectively. Tests were done twice at 5, 10 and 40 minutes, five times at 0 minute and 8 times at 15, 20, 25 and 30 minutes. Values are presented as the mean ± SE (Standard Error).

The concentration (y) of succinyl-CoA formed was evaluated using the calibration curve (y = 28564x + 35388; R^2^ = 0.9999), where peak area is x. The conditions under which the conversion of methylmalonyl-CoA into succinyl-CoA was linear with respect to incubation time and the amount of liver crude extract used were studied. The formation of succinyl-CoA increased linearly when the amounts of protein in the assay increased from 16 to 66 μg (Figure 2A). The rate of the formation of succinyl-CoA slowed down from 66 to 208 μg and even declined when a greater amount of protein was used (Additional file 3: Figure S2). The interconversion of methylmalonyl-CoA into succinyl-CoA, as illustrated in Figures 2B and Additional file 4: S3, followed a quadriphasic time course: an ascending phase (0-20 min), a slow-down phase (20-25 min), a plateau (25-30 min) and a decline (30-40 min). Hydrolysis or degradation of Coenzyme A (CoA) esters occurred during the process and Additional file 4: Figure S3 also shows the free CoA concentration. Both enzyme activity and hydrolysis decreased the amount of methylmalonyl-CoA during the time of incubation. Between 25 and 30 minutes, the concentration of methylmalonyl-CoA did not change drastically and was around 130 μM; accordingly, a reduction in the production of succinyl-CoA was not due to a depletion of methylmalonyl-CoA.

#### ***Specificity, recovery and analyte stability***

Specificity of the method was assessed by comparison of non-spiked samples with spiked ones. Both samples (non-spiked and spiked) had the same amount of protein. There was not any interfering peak from liver extract at the retention times corresponding to methylmalonyl-CoA or succinyl-CoA. The best recovery rate was observed using 66 μg of protein (Table 2). Stability of liver extract was studied to define optimal conditions for the analysis. Storage (frozen homogenate of fresh tissue or fresh homogenate of tissue snapped in liquid nitrogen) and processing conditions were studied. When homogenates were prepared from nitrogen-snapped frozen liver pieces, concentrations of the analyte were similar to those measured on homogenates prepared using fresh tissue and frozen at -80°C. The CV between the two types of homogenates was 8.21%. The analyte was also stable in liver crude extract for three frozen/thawed cycles when stored at -20°C and thawed to room temperature (CV = 7.30%). The % differences between two consecutive days (day 1 vs day 2; day 2 vs day 3) for fresh liver homogenates and homogenates of nitrogen-snapped liver tissue were 6.3; 7.7% and 5.8; 11.6% respectively. The inter-day CVs were 7.30 and 9.43%, respectively for the two types of homogenates. The quality control in each run reveals that the system is reliable with an accuracy of 88.76% (Table 2). As shown in Table 3, samples remained stable for at least 45 hours at room temperature with CVs varying from 4.55 to 9.22%. Similarly, when used three consecutive days, succinyl-CoA standard remained stable with an accuracy varying from 85% to 104% (Table 3). Data in Additional file 5: Table S2 strengthens the precision data of the method with accuracies varying from 84% to 93% for three control concentrations of methylmalonyl-CoA.

**Table 2:** Recovery and stability of succinyl-CoA according to sample storage condition

**Recovery data**					
**Level of protein tested (μg)**	**Level of standard added (μM)**	**n**	**Recovery%**	**CV%**	
33	75	4	73.79	12.61	
66	75	6	83.97	6.46	
166	75	3	70.83	14.72	
**Sample storage stability**					
	n	Mean Conc (μM)	CV%	CV% inter-day	% difference
Fresh liver					
day 1	5	60.88	1.72		day 1 vs day 2: 7.7
day 2	5	56.20	0.90		day 2 vs day 3: 6.3
day 3	5	52.64	3.25		day 1 vs day 3: 13.5
	15			7.30	
Nitrogen snapped Liver					
day 1	5	58.72	5.05		day 1 vs day 2: 11.6
day 2	5	51.93	8.21		day 2 vs day 3: 5.8
day 3	5	48.94	1.39		day 1 vs day 3: 16.7
	15			9.43	
Fresh vs Nitrogen	30	54.89		8.21	6
Control standard **75 μM**					Accuracy%
day 1	2	60.84	17.99		
day 2	2	69.80	10.82		
day 3	2	68.70	1.06		
day 4	2	64.69	0.51		
day 5	2	65.83	3.23		
day 6	2	69.57	6.84		
	12	66.57		8.18	88.76

The best recovery ratio was observed with 66 μg of protein. The results of storage stability correspond to the mean concentration of SSCoA (μM) formed by 66 μg of protein over 30 min of incubation at 37°C in presence of 200 μM of AdoCbl and 400 μM of MMCoA. Two vials of a known concentration of the standard (75 μM) were included as quality control.

**Table 3:** Stability of succinyl-CoA after completion of experiences and quality control test with succinyl-CoA standard solutions

**Post-process stability**					
**Sample ID**	**Injection count**	**Time after the first injection (hours)**	**Mean concentration of SSCoA (μM)**	**CV% inter-injection**	
Bovine-liver-1	2	33	33.74	9.22	
Bovine-liver-2	2	39	65.59	7.25	
Bovine-liver-3	2	40	56.11	6.67	
Bovine-liver-4	2	45	51.20	4.55	
**Standard (SSCoA)**		**High Qc**	**Middle Qc**	**Low Qc-2**	**Low Qc-1**
**Nominal value**		**500 μM**	**62.5 μM**	**7.8 μM**	**3.9 μM**
day 1					
		485.14	59.99	7.11	3.53
		415.51	63.46	7.12	3.98
		414.23	51.80	10.20	4.29
	Mean (n = 3)	438.29	58.42	8.15	3.93
	CV%	9.26	10.25	21.87	9.67
day 2					
		441.52	67.30	7.86	3.75
		435.70	56.49	7.97	3.72
		433.57	56.71	7.86	3.51
	Mean (n = 3)	436.93	60.16	7.90	3.66
	CV%	0.94	10.27	0.83	3.61
day 3					
		401.95	64.41	8.39	3.52
		405.45	64.14	8.48	3.67
		403.39	62.29	8.53	3.48
	Mean (n = 3)	403.60	63.61	8.47	3.56
	CV%	0.44	1.82	0.86	2.79
CV% inter-days (n = 9)		6.23	8.08	11.33	7.20
Accuracy%		85.25	97.17	104.75	95.29

The post-process stability study used 66 μg of protein over 30 min of incubation at 37°C in presence of 200 μM of AdoCbl and 400 μM of MMCoA. The quality control (Qc) study used four concentrations of the standard succinyl-CoA. The calculated accuracies varied from 85% to 104%.

### Pharmacokinetics

**Figure 3:**
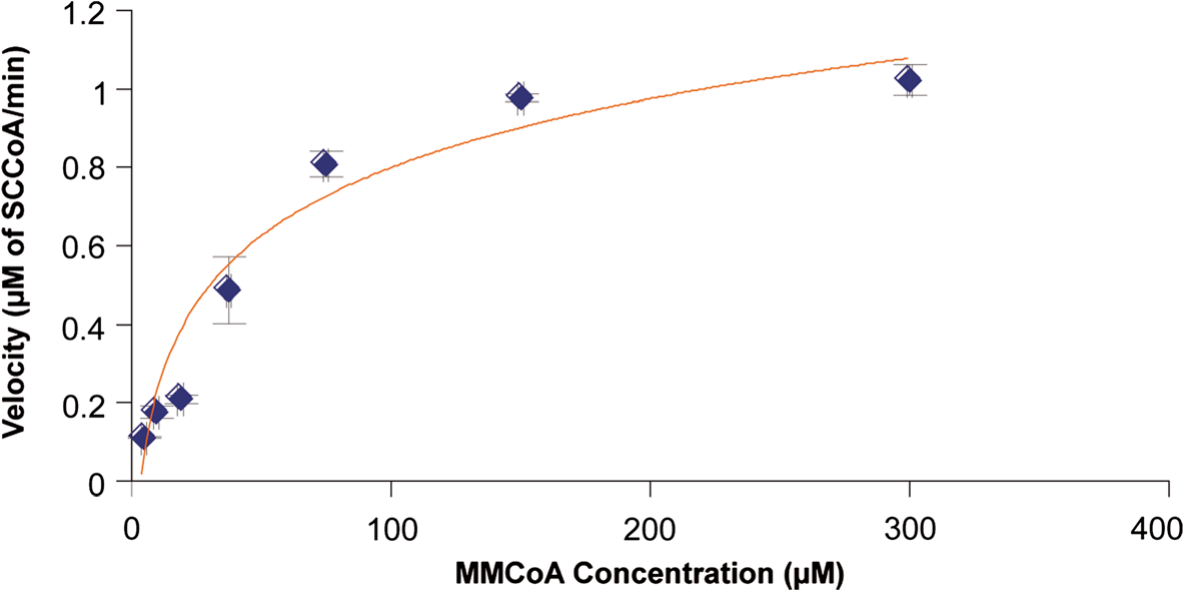
**Velocity of MCM versus increasing concentration of substrate after 30 min of incubation at 37°C.** The results are based on the mean concentration of succinyl-CoA (SCCoA) for 2 assays; we tested 7 substrate (methylmalonyl-CoA, MMCoA) concentrations (4.69-300 μM) using 66 μg of protein and 200 μM of AdoCbl. Each concentration level was duplicated in each assay. Error bars are the Standard Error (SE).

Figure 3 presents the velocity of MCM versus increasing concentration of substrate after 30 minutes of incubation at 37°C. Apparent kinetic parameters for MCM (Km and Vmax) were determined using 66 μg of protein and 200 μM of AdoCbl, but increasing concentrations of methylmalonyl-CoA (4.69-300 μM). Values for Vmax (1.1544 μM per min) and Km (49.126 μM) were calculated from Lineweaver-Burk representation and Eadie-Hofstee transformation of the data points respectively. When reported per mg of protein, Vmax was 17.5 μM of succinyl-CoA per minute per milligram of protein or 3.5 nmol/min/mg protein if the volume (200 μL) of reaction medium is taken into account. Mean specific activity of MCM in a bovine liver obtained from a slaughterhouse was 5.9 ± 0.4 nmol/min/mg protein (Table 4).

**Table 4:** Specific activity of MCM measured in liver obtained from slaughterhouse

**Runs**	**Total SA ± SD (n = 5) nmole/min/mg protein**	**CV% intra-run (n = 5)**	**CV% inter-run (n = 45)**
Run 1	5.563 ± 0.200	3.60	
Run 2	6.569 ± 0.333	5.07	
Run 3	6.015 ± 0.281	4.68	
Run 4	6.465 ± 0.378	5.85	
Run 5	6.234 ± 0.502	8.05	
Run 6	5.558 ± 0.166	2.98	
Run 7	6.150 ± 0.106	1.72	
Run 8	5.677 ± 0.051	0.90	
Run 9	5.317 ± 0.173	3.25	
Mean SA	5.950 ± 0.44		7.40

Assays were performed in presence of 200 μM of AdoCbl and 400 μM of MMCoA. Incubation was conducted at 37°C for 30 min. The amounts of protein used in the assays were 66 μg. Specific activities (SA) are expressed as nmoles of succinyl-CoA/min/mg protein. Standard deviation (SD) and coefficients of variation (CV%) are also presented.

The holomutase specific activity in liver of six lactating dairy cows at the same stage of lactation and fed the same diet represented between 2 and 5% of the total activity, it varied from 0.352 ± 0.002 to 0.68 ± 0.01 nmol/min/mg protein whereas the total specific activity varied from 12 ± 1 to 15.4 ± 0.6 nmol/min/mg protein (Table 5).

**Table 5:** Specific activity of MCM measured in liver of dairy cows at their peak of lactation

**Dairy cows**	**Total SA ± SD (n = 3) nmole/min/mg**	**CV%**	**Holo SA ± SD (n = 3) nmole/min/mg**	**CV%**
1	15.249 ± 0.543	3.56	0.467 ± 0.035	7.57
2	12.027 ± 0.973	8.09	0.561 ± 0.036	6.37
3	15.415 ± 0.631	4.10	0.489 ± 0.012	2.46
4	13.091 ± 0.397	3.03	0.513 ± 0.046	8.88
5	14.552 ± 0.881	6.05	0.352 ± 0.002	4.65
6	12.714 ± 0.405	3.18	0.676 ± 0.014	2.09

Assays were performed in presence of 200 μM of AdoCbl and 400 μM of MMCoA. Incubation was conducted at 37°C for 30 min. The amounts of protein used in the assays were 40 μg. Specific activities (SA) are expressed as nmoles of succinyl-CoA/min/mg protein. Standard deviation (SD) and coefficients of variation (CV%) are also presented.

## Discussion

Several methods for measuring MCM activity have been published. Some of these methods used radioactive isotopes [9-17], others are not [8,18-22]. Among these latter methods, evidence suggests that HPLC is more reproducible and accurate for measurement of small amounts of succinyl-CoA [29]. Validated data for the RP-HPLC according to criteria from FDA guidelines for bioanalytical method validation [32] are provided.

Bovine liver was chosen because of the importance of gluconeogenesis for ruminant animal, one of the major substrate being propionate [33]. A series of studies were performed to investigate the interconversion of methylmalonyl-CoA into succinyl-CoA in this tissue. As can be seen from this study, the amounts of succinyl-CoA formed increased with time until 20 minutes of incubation and the reaction is completed after half-hour of incubation. Conversion of methylmalonyl-CoA into succinyl-CoA also increased when increasing the amount of protein until 208 μg. It was also observed that reaction rates increased with increasing amounts of substrate. The method is specific; its reproducibility depends on the initial concentrations of the enzyme in the studied tissue. The reproducibility of the method published by Gaire and colleagues was greatly improved by bringing the inter-day CV from 12.16% [29] to 7.30% (present study). After completion of experiences, analyte remained stable and an analytical run could be reanalyzed in the case of instrument failure.

Analytes were considered stable in mouse [34] and human [35] plasma when the difference of concentration was less than 15% between freshly prepared samples and samples tested under various conditions [34,35]. In the present study, difference between fresh homogenates prepared from liver biopsies snapped in liquid nitrogen and frozen homogenates prepared from fresh tissue (6%) was inferior to 15%. Similarly, after 3 frozen/thawed cycles, this difference was low for two consecutive days. Accordingly, present results suggested that the analytes are stable during sample storage, extraction and chromatography processes for MCM in liver samples. However repeated frozen/thawed cycles of homogenates should be avoided because in spite of low CV, the average concentration numerically decreased from day 1 to day 3.

The estimated Km of the enzyme MCM calculated in the present study is lower than in previous reports. Willard and Rosenberg measured a Km value of 70 μM for the crude human MCM in fibroblast extracts [36]. A comparable Km (60 μM) has been measured by Taoka and co-workers with human recombinant MCM [7]. Higher values have been reported for human (300 to 350 μM) and mouse hepatic MCM (270 μM) using a radiolabeled methodology [37,38]. To determine MCM activity, Taoka et al. used the spectrophotometric detection of the CoA resulting from the hydrolysis of the succinyl-CoA. Results from the present study are lower than the mean Km value previously reported by our team (160 μM) in liver of lactating dairy cows [8], using the spectrometric assay method described by Taoka et al. [7]. Using a HPLC method, Gaire et al. measured a total specific activity of hepatic MCM in rat of 6.15 nmol/min/mg protein [29] whereas the radiometric method gave a value of 1.9 nmol/min/mg protein for the same sample [29]. The discrepancies observed may not only be species-dependent, especially ruminant versus non-ruminant, but also due to the method limitations.

Total specific activity in liver of dairy cows previously reported [8] using mitochondrial extract averaged 10.8 nmol/min/mg protein and was in the same range than the present data for lactating dairy cows. Total specific activity of MCM in a liver obtained from a slaughterhouse (5.95 nmol/min/mg protein) was similar to published values obtained in rat [29,30], mouse [38] and sheep [23] livers.

In ruminants, methylmalonyl-CoA is involved in gluconeogenesis [27]. Propionate is the major substrate for gluconeogenesis in dairy cows [6], and MCM is a key enzyme in the metabolism of propionate [39], essential to its entry into Krebs cycle and subsequent gluconeogenesis. Amongst all ruminants, efficient gluconeogenesis is most important in high-producing dairy cows because it is the major pathway for maintaining adequate glucose supply to the mammary gland for milk production [40]. In the present study, it was not known whether the liver bought from a slaughterhouse was from a steer, a heifer or a lactating or dry cow. Therefore, differences observed in specific activities between the liver obtained from the slaughterhouse and liver tissues obtained from lactating dairy cows at their peak of lactation are likely due to the physiological stage of the animal. Nevertheless, it cannot be ruled out that difference in the mode of preparation (treated within less than 1 hour after slaughter or frozen immediately after biopsy) could affect MCM activity measurements.

## Conclusions

Recently, a capillary electrophoresis method to measure MCM activity has been published [30]. We took an interest in that method for its short elution time (10 min) but the separation buffer needed 15 mM of sodium dodecyl sulfate (SDS) [30]. SDS is a highly effective surfactant [41]. In routine applications, dedication of one capillary to each application (particularly if surfactants are present) is recommended [42]. Furthermore, the high-concentration surfactant decreases both the separation efficiency and the reproducibility of the electrophoretic capillary method [31]. Indeed, as regards the signal-to-noise ratio of 3, the authors had a LOD of 3.4 μM [30] suggesting that present RP-HPLC is more sensitive with a LOD of 1 μM for the succinyl-CoA. As described in the materiel and method section, a centrifugation step after protein precipitation using TCA improved repeatability as compared to previous published results [29]. In addition, 100 mM of acetic acid in mobile phase improved the separation of residual substrate (methylmalonyl-CoA) and product (succinyl-CoA), and consequently contributed to improve system suitability. Finally the method is specific, accurate, precise and more sensitive than the previously published ones for the quantitative determination of methylmalonyl-CoA and succinyl-CoA in liver. It can be easily implemented into routine practice. This comprehensively validated method will allow studying the effects of stages of lactation, diet composition, and physiology in cattle. It is ideal for analysis of MCM activity and hence a tool to monitor cobalamin status in experimental and clinical studies.

## Abbreviations

RP-HPLC: Reversed-phase high performance liquid chromatography; CoA: Coenzyme A; MCM: Methylmalonyl-CoA mutase; AdoCbl: Deoxyadenosylcobalamin; TCA: Trichloroacetic acid; CV: Coefficient of variation; S/N: Signal-to noise ratio; LOD: Limit of detection; LLOQ: Low limit of quantitation; SDS: Sodium dodecyl sulfate; SSCoA: Succinyl-CoA; MMCoA: Methylmalonyl-CoA; SA: Specific activity; Qc: Quality control.

## Competing interests

The authors declare that they have no competing interests.

## Authors’ contributions

CLG participated to the design of the study and did a critical revision of the manuscript. BO designed the study, carried out the experiments, analysed the results and wrote the draft of the manuscript. MD provided biopsies of the lactating dairy cows and a critical revision of the manuscript. All authors read and approved the final manuscript.

## Supplementary Material

Additional file 1: Figure S1 Calibration curve of methylmalonyl-CoA. The linearity range was 3.9 to 500 μM. Area under of the curve (AUC) is presented as a function of methylmalonyl-CoA concentration (MMCoA Conc) expressed in μM. Click here for file

Additional file 2: Table S1 System suitability parameters. Values in the results column are calculated according to Figure 1. t1 = retention time of the peak of methylmalonyl-CoA. t2: retention time of the peak of succinyl-CoA. w1 = Width at half height for the peak of methylmalonyl-CoA. w2: width at half for the peak of succinyl-CoA. L: length of the column in centimetres. t0: time of unretained peak. tL: retention time of leading edge at 5% height. tT: retention time of tailing edge at 5% height. Click here for file

Additional file 3: Figure S2 Velocity for increasing amounts of protein after 30 minutes of incubation at 37°C. Concentration of succinyl-CoA formed (μM) per min according to the quantity of protein used in the assay (0 – 333 μg) in presence of 200 μM of AdoCbl and 400 μM methylmalonyl-CoA. Click here for file

Additional file 4: Figure S3 Changes in coenzyme A, succinyl-CoA and methylmalonyl-CoA concentrations according to incubation time. The results are mean concentrations of CoA (Coenzyme A), SSCoA (succinyl-CoA) and MMCoA (methylmalonyl-CoA) for 66 μg of protein incubated at 37°C. The initial concentration of AdoCbl and MMCoA were 200 μM and 263 μM respectively. Tests were done twice at 5, 10 and 40 minutes, five times at 0 minute and 8 times at 15, 20, 25 and 30 minutes. Inserted graph emphasizes the pattern of SSCoA. Click here for file

Additional file 5: Table S2 Stability of three concentrations of standard solutions of methylmalonyl-CoA. The quality control (Qc) study used three concentrations of the methylmalonyl-CoA measured in two assays during three consecutive days. The calculated accuracies varied from 84% to 93%. Click here for file
