# Supplementary figures and images for: Optimization and validation of a reversed-phase high performance liquid chromatography method for the measurement of bovine liver methylmalonyl-coenzyme a mutase activity
Source: BMC Biochem. 2013 Oct 16;14:25. doi: 10.1186/1471-2091-14-25 (PMC3856599; doi:10.1186/1471-2091-14-25)

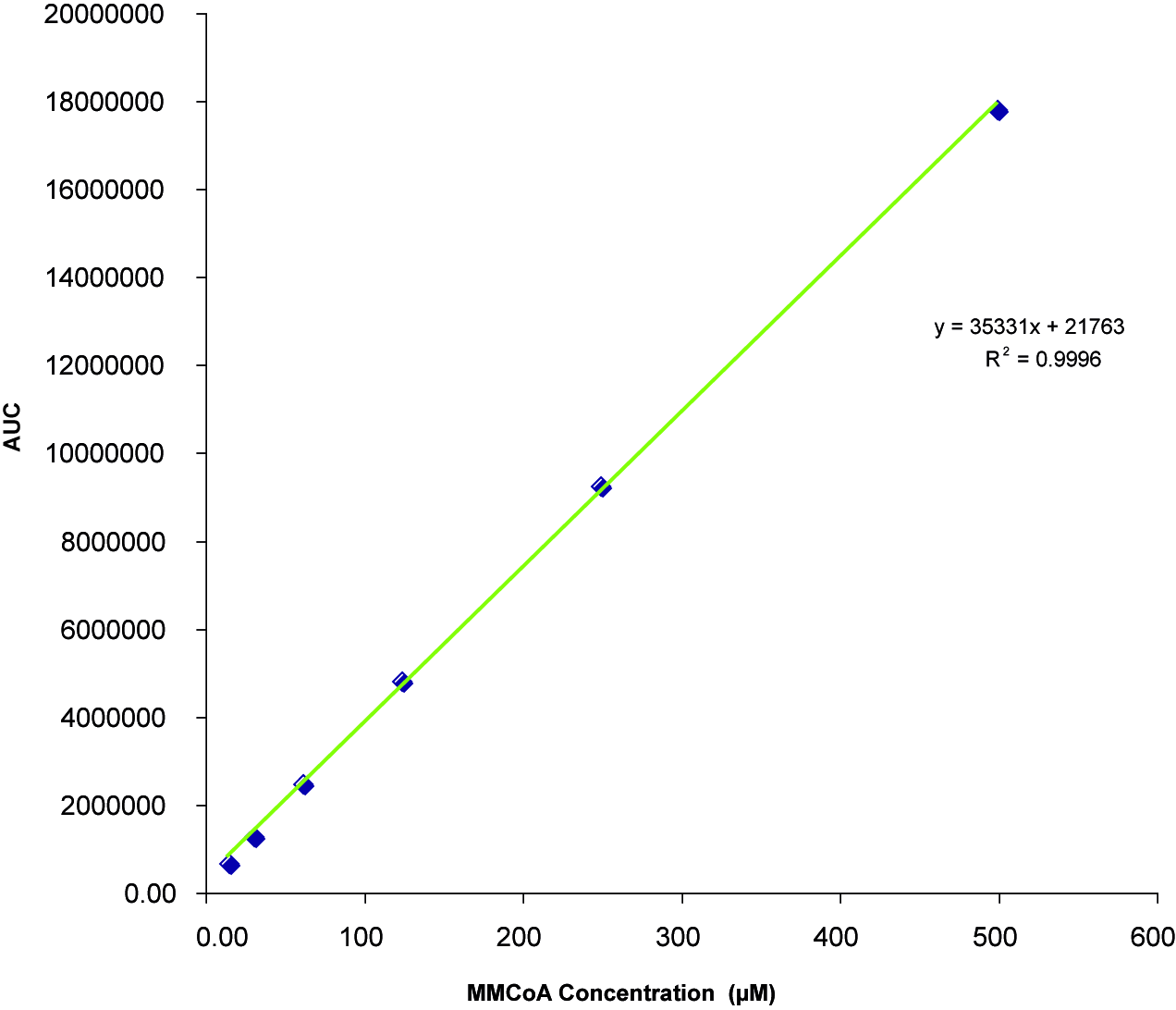

Supplement: Additional file 1: Figure S1 — Calibration curve of methylmalonyl-CoA. The linearity range was 3.9 to 500 μM. Area under of the curve (AUC) is presented as a function of methylmalonyl-CoA concentration (MMCoA Conc) expressed in μM. [file 1471-2091-14-25-S1.tiff]

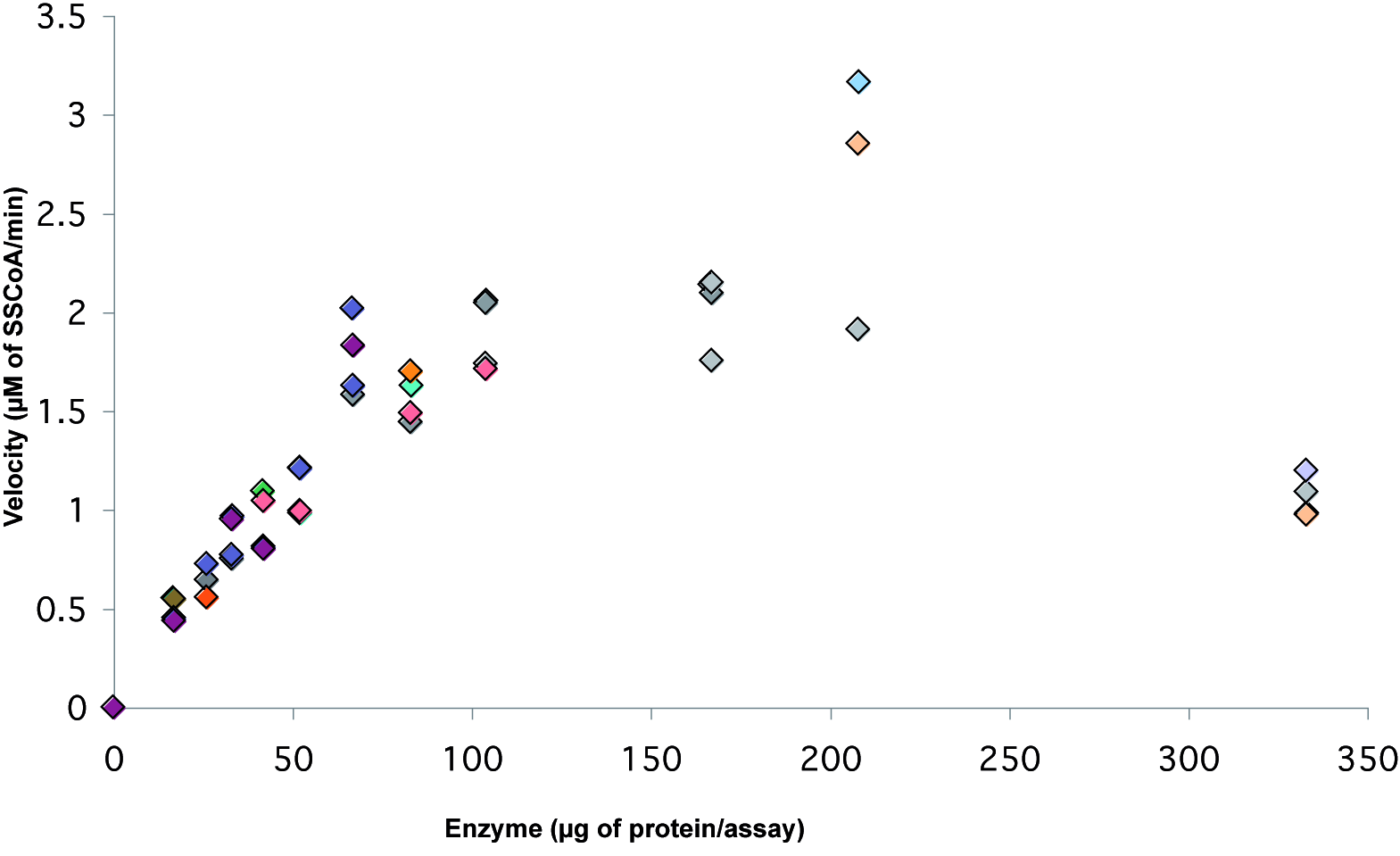

Supplement: Additional file 3: Figure S2 — Velocity for increasing amounts of protein after 30 minutes of incubation at 37°C. Concentration of succinyl-CoA formed (μM) per min according to the quantity of protein used in the assay (0 – 333 μg) in presence of 200 μM of AdoCbl and 400 μM methylmalonyl-CoA. [file 1471-2091-14-25-S3.tiff]

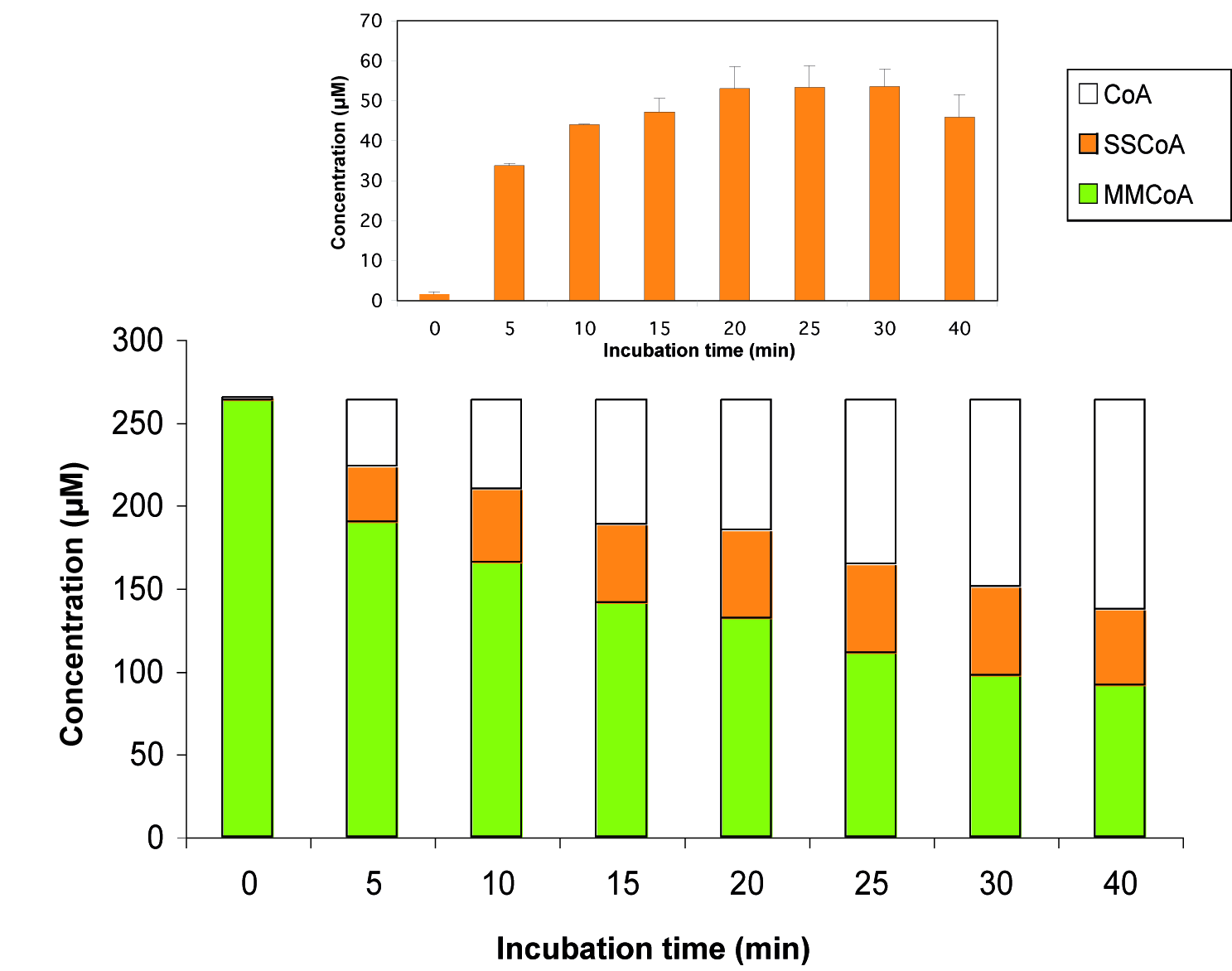

Supplement: Additional file 4: Figure S3 — Changes in coenzyme A, succinyl-CoA and methylmalonyl-CoA concentrations according to incubation time. The results are mean concentrations of CoA (Coenzyme A), SSCoA (succinyl-CoA) and MMCoA (methylmalonyl-CoA) for 66 μg of protein incubated at 37°C. The initial concentration of AdoCbl and MMCoA were 200 μM and 263 μM respectively. Tests were done twice at 5, 10 and 40 minutes, five times at 0 minute and 8 times at 15, 20, 25 and 30 minutes. Inserted graph emphasizes the pattern of SSCoA. [file 1471-2091-14-25-S4.tiff]
