# Supplementary material for: Optimization and validation of a reversed-phase high performance liquid chromatography method for the measurement of bovine liver methylmalonyl-coenzyme a mutase activity
Source: BMC Biochem. 2013 Oct 16;14:25. doi: 10.1186/1471-2091-14-25 (PMC3856599; doi:10.1186/1471-2091-14-25)
Supplement: Additional file 2: Table S1 — System suitability parameters. Values in the results column are calculated according to Figure 1. t1 = retention time of the peak of methylmalonyl-CoA. t2: retention time of the peak of succinyl-CoA. w1 = Width at half height for the peak of methylmalonyl-CoA. w2: width at half for the peak of succinyl-CoA. L: length of the column in centimetres. t0: time of unretained peak. tL: retention time of leading edge at 5% height. tT: retention time of tailing edge at 5% height. [file 1471-2091-14-25-S2.doc]

**Additional file 1: Table S1 System suitability parameters**

| Calculation | Formula | Results | Recommendations |
| --- | --- | --- | --- |
| Theoretical plates (N) | N=5.5452(t1/w1)^2^ | N = 14480 | N > 2000 |
| Capacity factor (k’) | k’=(1/t0)-1 | k1’ = 10 | k’ > 2 |
|  |  | k’2 = 12 |  |
| Selectivity factor (α) | α=k’2/k’1 | α = 1.21 | α > 1 |
| Resolution (R) | R=2(t2-t1)/(1.699(w1+w2)) | R = 6.04 | R > 2 |
| Peak tailing 5% (T) | T=(tT-tL)/(2(t1-tL) | T1 = 1.30 | T ≤ 2 |
|  |  | T2 = 1.36 |  |

Values in the results column are calculated according to Figure 1. t1=retention time of the peak of methylmalonyl-CoA. t2: retention time of the peak of succinyl-CoA. w1=Width at half height for the peak of methylmalonyl-CoA. w2: width at half for the peak of succinyl-CoA. L: length of the column in centimetres. t0: time of unretained peak. tL: retention time of leading edge at 5% height. tT: retention time of tailing edge at 5% height.
