# Supplementary material for: Optimization and validation of a reversed-phase high performance liquid chromatography method for the measurement of bovine liver methylmalonyl-coenzyme a mutase activity
Source: BMC Biochem. 2013 Oct 16;14:25. doi: 10.1186/1471-2091-14-25 (PMC3856599; doi:10.1186/1471-2091-14-25)
Supplement: Additional file 5: Table S2 — Stability of three concentrations of standard solutions of methylmalonyl-CoA. The quality control (Qc) study used three concentrations of the methylmalonyl-CoA measured in two assays during three consecutive days. The calculated accuracies varied from 84% to 93%. [file 1471-2091-14-25-S5.doc]

**Additional file 5:** Table S2 Stability of three concentrations of standard solutions of methylmalonyl-CoA

| **Standard** |  | **High Qc** | **Middle Qc** | **Low Qc** |
| --- | --- | --- | --- | --- |
| **Nominal value** |  | **500 μM** | **62.5 μM** | **3.9 μM** |
| day 1 |  |  |  |  |
|  |  | 496.93 | 63.92 | 3.60 |
|  |  | 430.87 | 56.64 | 3.99 |
|  | Mean (n=2) | 463.90 | 60.28 | 3.80 |
|  | CV% | 10.07 | 8.54 | 7.20 |
| day 2 |  |  |  |  |
|  |  | 428.11 | 56.95 | 2.63 |
|  |  | 484.24 | 57.98 | 3.24 |
|  | Mean (n=2) | 456.17 | 57.46 | 2.94 |
|  | CV% | 8.70 | 1.27 | 14.73 |
| day 3 |  |  |  |  |
|  |  | 477.96 | 58.18 | 3.20 |
|  |  | 481.04 | 58.12 | 3.09 |
|  | Mean (n=2) | 479.50 | 58.15 | 3.14 |
|  | CV% | 0.45 | 0.07 | 2.48 |
| Accuracy % |  | 93.30 | 93.81 | 84.40 |
| CV% inter-days (n=6) | | 6.31 | 4.55 | 14.11 |

The quality control (Qc) study used three concentrations of the methylmalonyl-CoA measured in two assays during three consecutive days. The calculated accuracies varied from 84 % to 93%.
